# Adaptation of patients diagnosed with human papillomavirus: a grounded theory study

**DOI:** 10.1186/s12978-021-01264-y

**Published:** 2021-10-26

**Authors:** Narjes Nick, Camellia Torabizadeh, Mehdi Ghahartars, Roksana Janghorban

**Affiliations:** 1 Department of Nursing, School of Nursing and Midwifery, Community Based Psychiatric Care Research Center, Shiraz University of Medical Sciences, Shiraz, Iran; 2 Molecular Dermatology Research Center, Shiraz University of Medical Sciences, Shiraz, Iran; 3 Department of Midwifery, School of Nursing and Midwifery, Community Based Psychiatric Care Research Center, Shiraz University of Medical Sciences, Shiraz, Iran

**Keywords:** Human papillomavirus infection, Adaptation, Psychological, Grounded theory, Qualitative research

## Abstract

**Background:**

Human papillomavirus is the most common cause of sexually transmitted diseases. Various studies report that positive human papillomavirus diagnosis results in psychosexual issues for the infected and reduces their quality of life. However, the adaptation of the infected has not been addressed yet. The present study aims to identify the process by which individuals infected with human papillomavirus adapt to their disease.

**Method:**

This is a qualitative work of research with a grounded theory design. The setting of the study was the skin clinic of Shahid Faghihi Hospital in Shiraz. The participants consisted of 27 individuals: 18 patients, 3 doctors, 2 counselors, and 4 spouses of patients. The subjects were selected via purposeful and theoretical sampling method until data saturation was reached. Data were collected through face-to-face, in-depth, semi-structured interviews from April 2019 to December 2020. The collected data were analyzed using Corbin and Strauss’s method (2015) and MAXQDA 2018.

**Results:**

The theory which emerged from the data was “trying to maintain resilience in the absence of psychological security.” Analysis of data showed the main concern of participants in adapting to their diagnosis with human papillomavirus was “life stress”. “Stigma and ignorance” was found to be a contextual condition and “paradox in support” was an intervening condition in the patients’ adaptation. The patients’ action/interaction responses to their main concern in the context in question were “emotional confrontation” and “maintaining resilience.” The outcome was “oscillation between tension and tranquility.”

**Conclusion:**

The present study explains the process by which patients with human papillomavirus adapt to their condition. Identification of the concerns of patients with human papillomavirus and the factors which affect their adaptation can help healthcare policy-makers and providers develop effective support plans in order to increase patients’ quality of life. Early interventions, e.g. counseling care providers to modify their behaviors toward alleviating the psychosexual tension of the infected, can facilitate the adaptation of the infected and decrease the consequences of the infection for them.

**Graphical Abstract:**

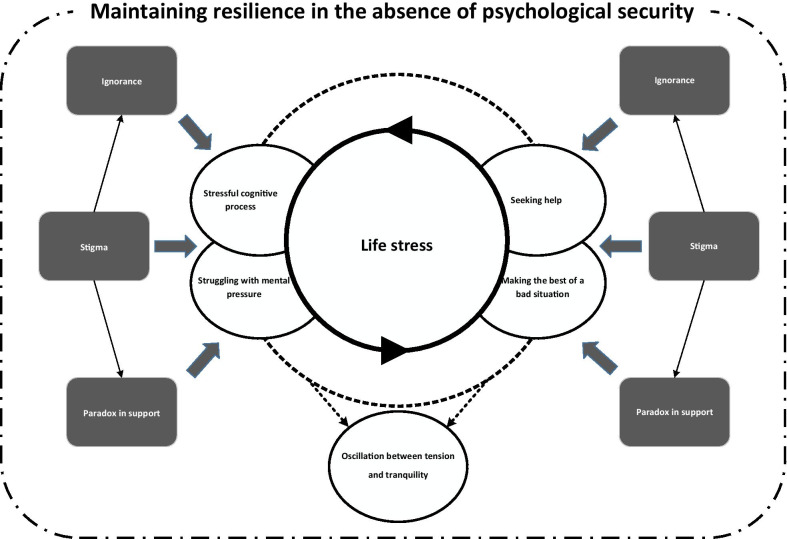

## Plain language summary

Human papillomavirus (HPV) is the most common cause of sexually transmitted diseases. Almost all men and women get the infection at least once throughout their lives. The high-risk types of HPV account for about 5% of cancer cases globally. HPV can cause anogenital cancers and warts in both genders. In this grounded theory study, we conducted 27 in-depth interviews with Iranian patients, their spouses, and health care providers from April 2019 to December 2020. The collected data were analyzed using Corbin and Strauss’s method (2015) and MAXQDA 2018. The participants were concerned about tension in family relationships, being stigmatized, getting cancer, recurrence of warts, transferring their disease to others, and changes in the appearance of their genitalia. They stated that HPV is regarded as a shameful disease in society. Most of the participants said they had never heard anything about HPV. The patients’ action/interaction responses to their concerns were “emotional confrontation” and “maintaining resilience.” These strategies helped the patients recover some of their tranquility. However, some of patients’ concerns were persistent and kept them oscillating between tension and tranquility. An understanding of the patients’ perception of their disease is essential to development of effective educational interventions to change patients’ perspective on their situation and improve their recovery. Furthermore, because of the low level of public awareness about HPV and sexual health and the flow of misinformation to the infected, it is recommended that educational interventions focus on the patients’ concerns.

## Introduction

Human papillomavirus (HPV) is the most common cause of sexually transmitted diseases (STDs). Almost all men and women get the infection at least once throughout their lives [1]. The high-risk types of HPV account for about 5% of cancer cases in the world, and approximately 570,000 women and 60,000 men contract HPV-related cancers every year [2].

Adaptation to the disease is a complex, dynamic, periodic, and interactive process. This process is influenced by a variety of factors, including one’s coping strategies, personal characteristics, values, beliefs, and life experiences [3, 4], as well as the characteristics of the infection, underlying factors, and social support, all of which are interrelated [5, 6]. Also, adaptation is influenced by culture [5]. There is an unspoken nature of sexuality in Iranian culture [7] since in this culture, sexual matters are regulated by limitations, taboos, and unwritten rules [8]. Successful adaptation to HPV requires comprehensive physical and psychological adaptation, and an understanding of this process entails an investigation of the underlying factors [9].

A review of literature shows that the studies conducted on HPV are mostly focused on women, and such matters as quality of life [10], marital adaptation to the infection (using a quantitative approach) [11], vaccination [12], the psycho-sexual effects of being diagnosed with HPV [13, 14], women’s experiences of facing their infection [15], women’s psychological responses to positive HPV test [16], the information management processes of women living with HPV [17], and reproductive concerns of HPV- positive women [18]. Research findings show that testing positive for human papillomavirus is accompanied by feelings of shame, stigma, self-deprecation, regret, anxiety and concern [16, 18, 19]. To the best knowledge of the authors, no work of research has addressed the process of adaptation in men and women with HPV. Only one work of research has addressed the psychosocial adaptation of women diagnosed with HPV. According to this study, the sexual and psychological adaptation of women diagnosed with HPV was achieved after 6 months. But, this study does not deal with the coping strategies and conditions of the infected and does not conduct an in-depth analysis of the experiences of the infected due to its quantitative approach [20]. A study of this process can help identify patients’ main concerns, their coping strategies and the factors affecting their adaptation, and this information can be used by healthcare policy-makers. Also, nations worldwide are developing plans to promote global health, which requires relevant and context-sensitive evidence to support their policies and interventions [21]. Thus, we proposed the following research question: “How patients diagnosed with HPV adapt to their diagnosis?” and the aim of the present study was to explore this adaptation process.

## Method

### Study design

The present study is a qualitative work with a grounded theory design, conducted from April 2019 to December 2020. Grounded theory answers the question of how individuals respond to events or handle problems through action and interaction [22].The setting of the study was the skin clinic of Shahid Faghihi Hospital in Shiraz. It is a referral center for patients with HPV diagnosis with or without skin manifestation in the south of Iran. Twenty-seven people participated in the study: 18 patients, 3 doctors, 2 counselors, and 4 spouses of patients. At first, subjects were selected via purposeful sampling. Next, based on the concepts and categories extracted from the initial data, the researchers employed theoretical sampling to collect more data. Analyses of the first 8 interviews raised questions about the reactions of the spouses of patients with HPV. Accordingly, interviews 9, 13, 24, and 26 were conducted with the spouses of 4 patients with HPV. As all of the participants referred to the role of healthcare providers, 5 interviews were conducted with the members of this population. To learn about as many viewpoints as possible, the researchers applied maximum variation sampling in terms of age, gender, virus type, occupation, education, length of infection, marital status, number of children, and type of sexual relationship. The healthcare providers were selected from both the public and private sectors. The inclusion criteria for the patients were having been diagnosed with HPV as confirmed by a doctor’s certificate or having positive HPV test results and willingness to participate in the study. The exclusion criteria were having cancer, HPV-related cancer, Low and High Grade Squamous Intraepithelial lesions or a different type of sexually transmitted disease. Only one of the patients at the clinic refused to participate in the study due to professional reasons.

The patients’ trust in the first author was enhanced by the following factors: the doctors’ trust in the researcher, the researcher’s regular presence at the clinic, answering the patients’ questions and providing them with reliable information, the researchers’ affiliation with a university, the doctors’ referring patients to the researcher, and the researcher’s physical presence in the clinic in a room of her own. In addition, the patients had been informed that refusal to participate in the study would not affect the process of their treatment.

### Data collection method

Data were collected through individual, face-to-face, in-depth, semi-structured interviews. The interviews were conducted by N.N. and each lasted from 30 to 100 min. Most of the interviews were conducted in a quiet room at the clinic and were recorded on a voice recorder. 3 of the participants who did not feel comfortable about being interviewed at the clinic were interviewed at places of their choice. Due to the spread of the coronavirus, participant 18 was interviewed online. The healthcare providers were interviewed at their workplace. At the time of each interview, the interviewer and the participant were alone. One of the participants was interviewed twice for further clarification of some points raised during the first interview. Thus, in total, 28 interviews were conducted.

Each interview with the patients started with a few general, open-ended questions related to the main research objective followed by questions which casually came up in the course of the interviews. The first question was, “Can you tell me about the first time when you found out you had the infection?” One of the main questions was, “What were your experiences when you told others about the result of your diagnosis?” Based on the participants’ answers, exploratory questions were asked: “Can you explain that more?” or “Can you give an example?” The concluding question was, “Is there anything else you would like to add or talk about something I might have missed?”.

One of the questions which the spouses of the patients were asked was, “Can you tell me about the first time when your spouse talked to you about his/her infection?” An example of the questions which the healthcare providers were asked is, “What are your experiences of the reactions of patients or their companions when they are told that they have the infection?” Data collection continued until the data was saturated. In the present study, field note was also taken alongside the interviews and they were coded in the process of data analysis.

### Data analysis

The collected data were analyzed according to Corbin and Strauss’ method (2015). The stages of data analysis consisted of: open coding, determining concepts, developing the concepts and determining their characteristics and dimensions, analyzing data for context, bringing the process into the analysis, and integrating the categories [22]. Soon after the first interview, the process of data analysis began. Immediately after conducting each interview, N. N. listened to the recording carefully and transcribed it word by word. Subsequently, the handwritten transcripts were typed and compared to the audio files. Then the scripts were entered into MAXQDA 2018 and the open coding was performed. All the authors participated in the process of data analysis. The data were collected, coded, and analyzed simultaneously. Throughout the study, the researchers applied such analytical methods as constant comparison, questioning and waving the red flag. Also, the researchers used memos throughout the analytical process.

The credibility of the data were ensured using Corbin and Strauss’s criteria [22]. Prolonged engagement, maximum variation, peer checking, member checking, external auditing, and complete description of the participants and research settings were used to confirm the data credibility. Also, the experiences of C. T. and R. J. in the field of grounded theory enhanced the credibility of the research findings. The researchers were careful to avoid bias and letting their predetermined ideas affect the data. The interview transcripts and parts of the findings were given to the participants who were asked to confirm them.

## Research team and reflexivity

This article is part of the Ph.D. thesis of First author. C T. and R. J. were experienced in grounded theory projects. Also, the professional field of all the authors was related to the research topic, which enhanced the theoretical sensitivity of the study. The research process was carried out directly by the researchers. The research team consisted of man and woman members, all of whom had training and experience in research and publication. There was no relationship or conflict of interest between the researchers and participants. The participants were informed about the research topic and objectives before being interviewed.

### Ethical considerations

The present research has been approved by the ethics committee of Shiraz University of Medical Sciences, registered at IR.SUMS.REC.1398.431. All the participants filled out the informed consent form. For ethical considerations, before being interviewed, the participants were informed about the research objectives, the voluntary basis of their participation, their right to withdraw, the confidentiality of their information, and the use of a voice recorder during the interviews.

## Results

The age range of the patients was 19 to 50 years. 66.7% of the patients were married. Tables 1 and 2 show the other demographic characteristics of the participants.

**Table 1:** The demographic characteristics of the patients

No	Age	Gender	Education	Occupation	Marital status	Number of children	Length of infection (months)	Interval between symptom onset and diagnosis (months)	Virus type	Genital warts	Type of sexual relationship
1	33	Man	Associate degree	Office clerk	Married	1	84	60	Unknown (Spouse’s type was 18)	Yes	Vaginal
2	27	Man	Master’s degree	Self-employed	Single	0	10	2–3	Unknown	Yes	Vaginal/anal/oral
3	38	Man	Master’s degree	Self-employed	Married	0	120	6	Unknown	Yes	Vaginal
4	40	Woman	High school diploma	Self-employed	Married	3	6	0	16, 54	Yes	Vaginal/anal/oral
5	34	Woman	High school diploma	Homemaker	Married	2	8	0	45	Yes	Vaginal
6	34	Woman	Master’s degree	Homemaker	Married	1	1	0	6	Yes	Vaginal/anal/oral
7	19	Woman	High school diploma	Homemaker	Single	0	1	2	Unknown	Yes	Anal
8	50	Man	Middle school	Retired	Married	2	21	2	Unknown	Yes	Vaginal/anal/oral
9	27	Man	Bachelor’s degree	Unemployed	Single	0	36	14	Unknown	Yes	Vaginal/anal/oral
10	24	Woman	High school diploma	Unemployed	Single	0	1 week	0	6, 11, 53	Yes	Anal
11	43	Man	High school	Self-employed	Married	2	5	2	Unknown	Yes	Vaginal
12	28	Woman	Middle school	Prostitute	Married	2	2 weeks	0	^a^Positive non16 and 18	Yes	Vaginal/anal
13	32	Man	Bachelor’s degree	Office clerk	Married	0	12	2	11	Yes	Vaginal
14	40	Woman	High school diploma	Homemaker	Married	3	1	0	16	No	Vaginal/oral
15	30	Man	High school diploma	Self-employed	Single	0	10	1	Unknown	Yes	Vaginal/anal/oral
16	45	Man	Middle school	Self-employed	Married	2	5	4	Unknown (Spouse’s type was 6)	Yes	Vaginal
17	45	Woman	Middle school	Homemaker	Married	2	4	1	6, 66	Yes	Vaginal/anal/oral
18	32	Woman	Ph.D	Office clerk	Married	0	12	3	58	Yes	Vaginal

^a^To reduce the costs of medical tests or because of the policies of some doctors to reduce their patients’ stress, some patients run this kind of typing test in which the test kit only detects types 16 and 18. If a patient is infected, but his/her type is not 16 or 18, the test result comes out as “positive, non 16, 18.”

**Table 2:** The demographic characteristics of the healthcare providers and the patients’ spouses

No.	Gender	Age	Occupation	Work experience (yrs.)	Education
1	Woman	39	Counselor/midwife	11	Bachelor’s degree
2	Woman	37	Counselor/psychologist	14	Master’s degree
3	Woman	69	Doctor	45	OB & GYN, Gynecology Oncology
4	Man	50	Doctor	25	General practitioner
5^a^	Woman	45	A patient’s wife-homemaker	–	High school diploma
6^a^	Woman	38	A patient’s wife-homemaker	–	High school diploma
7^b^	Woman	32	A patient’s wife-office clerk	–	Bachelor’s degree
8	Man	43	Doctor	7	Dermatologist
9^a^	Man	36	A patient’s husband-self-employed	–	Bachelor’s degree

^a^At the time of the interviews, these patients’ spouses did not have any symptoms of HPV and had not run any diagnostic tests, so their infection status was undetermined

^b^These patients’ spouses had tested negative for HPV

The number of codes extracted from the 28 interviews was 4098 which fell to 1207 after the repeated codes were merged. Data reduction continued with constant comparison and interaction with the data. At first, 136 sub-sub-subcategories were extracted which were finally reduced to 79. As the analytical process went on, the labels which were related to the same concept were put together to form conceptual categories on higher abstraction levels. At the end of the process of concept generation, 27 sub-subcategories, 12 subcategories, and 6 categories emerged. The categories abstracted from data analysis are shown in Table 3.

**Table 3:** The subcategories, categories and core category

Subcategory	Category	Core category
Stigma	Context of stigma and ignorance	Maintaining resilience in the absence of psychological security
Poor awareness		
Sense of chaos in life	Life stress	
Sense of insecurity in life		
Supportiveness	Paradox in support	
Lack of support		
Stressful cognitive process	Emotional confrontation	
Struggling with mental pressure		
Seeking help	Maintaining resilience	
Making the best of a bad situation		
Mental exhaustion	oscillation between tension and tranquility	
Achieving relative tranquility		

The aim of the present study was to explore the process of adjustment of patients who tested positive for HPV. The main concern of the patients was found to be life stress. They repeatedly referred to experiencing stress in their lives. Their concern developed in the context of stigma and ignorance and under the influence of paradox in support. The patients used various coping strategies to deal with their issues. Overreacting, mental rumination, changing the truth, mental tension, obsessive behaviors, and social isolation were the strategies which were used initially. For fear of stigma or creating tension at home, some of the patients deprived themselves of the emotional support of the people around them. Yet, the others sought emotional support from their families, friends and healthcare providers. Other strategies used by the patients were boosting their tolerance and changing their lifestyle to improve an unpleasant situation. These strategies were not employed separately and in a linear manner, but were interrelated and sometimes, following a change in conditions, appeared simultaneously.

**Fig. 1:**
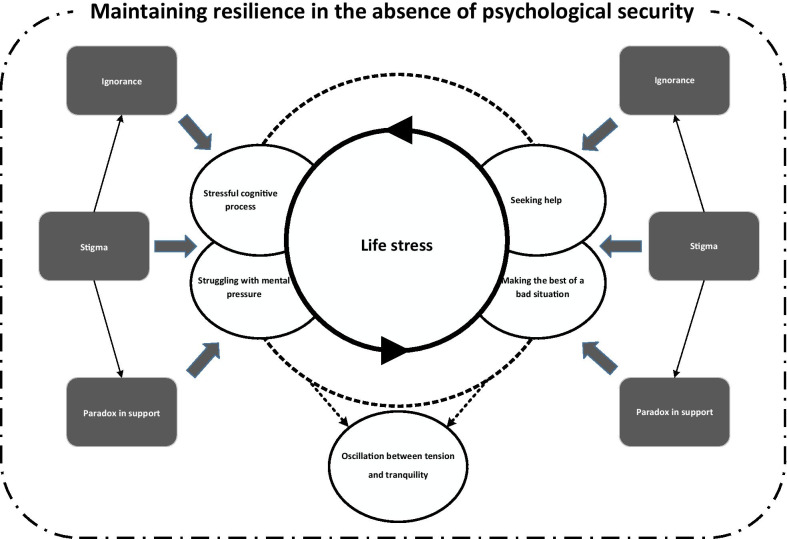
The theory of “trying to maintain resilience in the absence of psychological security” (the dotted circle shows the connection between strategies)

Overall, their coping strategies to manage their concerns took the form of emotional confrontation and trying to maintain resilience. The participants mentioned that the strategies they applied enabled them to have relatively normal lives, but any reference to their infection would make them tense again. Considering the frequent changes which they experienced and their many concerns, the participants were inclined toward adaptation, but oscillated between tension and tranquility in the process. In the present study, based on the participants’ behaviors and the results of the categorization process, “trying to maintain resilience in the absence of psychological security” was selected as the core category which connected all the categories and concepts extracted from the collected data. The theory which emerged from the analysis of data is shown in Fig. 1. In the following sections, the details of this process will be presented.

### Main concern: life stress

The dimensions of this concept are “having a sense of chaos in life” and “having a sense of insecurity in life.”

#### Having a sense of chaos in life

The participants mentioned that following their diagnosis, their spouses lost their psychological balance (e.g. they became aggressive, cried frequently, or got depressed).

My wife was really upset, she cried all the time. She was a different person. Our life was so stressful. We were always arguing with each other. (Participant 3, patient).

The tension in marital relationships had resulted in many issues for the couples, ranging from a temporary lack of communication to emotional divorce and actual divorce in some cases. The tension affected not only the marital relationships, but the relationships between the other members of the families.

Things were a mess at home …. The kids would cry and tell me, ‘You’re a bad mom. We wish you weren’t our mom.’ (Participant 5, patient).

Concern about the spread of the infection in the family and the issue of unfaithfulness were among the reasons which the participants mentioned to explain the chaotic atmosphere in their families.

#### Having a sense of insecurity in life

One of the concerns of all the participants was fear of being stigmatized by the society and their families. They were worried that if others found out about their unlawful relationships, they would face disgrace, be called names, and be ostracized.

Others would call us immoral people, they would say we must have been having affairs. (Participant 16, patient).

The participants stated that because the disease was infectious, they were worried about transferring the infection to their sexual partners. They also expressed their concern about infecting their future spouses and their babies if they became pregnant. One of the participants said, *I was thinking if I get married now, I will give the infection to my husband. Or if I want to have a baby, it will affect my baby* (Participant 10, patient).

In addition, the knowledge that they have HPV and that the virus can cause cancer results in the infected being haunted by fear of cancer and even death.

When my test result showed it was malicious, I told my husband I felt terrible. When he asked why, I said they told me it’s malicious. I’m afraid of cancer. (Participant 17, patient).

The patients were also worried about recurrence of their genital warts, the possibility of carrying the virus even after recovery and having the infection as a chronic disease, and changes in the appearance of their genitalia because of the warts or scars left by treatment. One of the participants said, *I’m scared. This disease is incurable and the virus will be in my body forever.* (Participant 6, patient).

### Data analysis for context

Context is a combination of all the circumstances that constitute a situation and includes the patients’ reasons for their interactions. In the present study, the contextual condition of a context of stigma and ignorance, which included the socio-political conditions which had influenced the patients’ experiences, and the intervening condition of paradox in support were perceived as facilitators or inhibitors in the process of adaptation [22].

#### Context of stigma and ignorance

The participants stated that extramarital sexual relationships are unacceptable to the society and family and are regarded as taboo, and personal, social, and family beliefs are affected by this taboo. The fact that HPV is regarded as a shameful disease.

This disease is like a label which is attached to you and never comes off. (Participant 8, patient).

In some cases, the patients were frequently asked questions about how they had been infected by the people around them who were curious about their disease. The participants mentioned that the cultural taboo about discussing sexual matters prevents adolescents from receiving systematic and continuous education about puberty and sexual relationships and continue to engage in high-risk behaviors. One of the participants said, *I didn’t know much about safe sex. If I had, I wouldn’t be here now.* (Participant 2, patient).

It was also pointed out that in the absence of proper sex education, the role of the family becomes very important. In the present study, only one of the participants was found to have received this kind of education from his family. The rest of them had started their sexual relationships with ignorance about how to lead a healthy sex life. In addition, the authorities’ inaction about HPV has resulted in public ignorance about the disease. Many of the participants complained that the healthcare system was keeping quiet about the disease and was not using the media to inform the public.

I’m 33 years old now. I had heard people talk about human immunodeficiency viruses (HIV) and hepatitis many times, but I’d never heard anything about HPV. (Participant 1, patient).

The participants mentioned that lack of awareness about the nature of HPV results in people’s not taking preventive or curative measures, including being vaccinated, practicing safe sex following infection, and seeking medical attention for their genital warts at a healthcare center before it is too late. Many of the participants attributed their delay in seeking medical attention to their unawareness about the infection. One of the doctors said, *Most patients don’t have much information about this infection. They often mistake their warts for moles and are late in seeking treatment.* (Participant 4, doctor).

Participants mentioned that the content of social media, including websites on the Internet and Instagram, on diseases is uploaded by individuals many of whom do not have any specialized knowledge of medicine. Also, because much of this content is out-of-date and inconsistent, it adds to patients’ concern. Many of the participants stated that the social media presents an exaggerated picture of cancer associated with HPV, occasionally compares HPV to HIV, and misinforms them about available treatments, all of which adds to their anxiety.

One of my concerns is the information patient find on Google. Much of what it says there is related to cancer. You don’t find much about differences between various types of viruses and their impacts on developing a particular cancer. (Participant 1, counselor).

#### Paradox in support

“A number of participants reported the experience of receiving support. The sources of this supportiveness included family, friends, and health service providers. One of the doctors said, *I tell my patients not to worry as the virus often clears up. The virus certainly disappears at ages below 30 years, leaving no causes for concern.* (Participant 3, doctor)” [23]. However, in some cases they experienced lack of support from relatives and health service providers. “One of the participants said, *We are truly alone in life. Right now, there is no one around and this makes the problem worse.* (Participant 3)” [23]. In fact, Patient’s’ interactions with different people resulted in different and, in many cases, contradictory experiences for them. The support which the infected receive from the people close to them (including the family and friends) and healthcare providers is an intervening condition in the adaptation process of patients with HPV [23].

### Data analysis for process

The patients’ action/interaction strategies with regard to stress in life in the context of stigma and ignorance fell into two categories: “emotional confrontation” and “trying to maintain resilience.”

#### Emotional confrontation

The concept of emotional confrontation consists of “stressful cognitive process” and “struggling with mental pressure.”

##### Stressful cognitive process

The patients’ mental picture of their disease as a monster or terrible disaster stems from stress in life under the conditions. One of them mentioned, *My child was only 3. She died in my own arms. It was very painful. I don’t know what to say. I just know that, yeah, my child’s death was terrible, but when I was told I had this infection, it felt worse. (The patient cried at this point). I wish I had a heart disease or MS or had become paralyzed instead of getting this disease.* (Participant 4, patient).

In addition to stress in life, contextual conditions and the treatment teams’ interactions with the patients aggravated the patients’ tension. A few of the participants referred to the social media as the main cause of their anxiety and need to see a counselor. Some of the patients mentioned that after their diagnosis, they kept comparing themselves with other patients and tried to remember their relatives’ experiences of cancer or HIV—they said that the thought of their disease haunted them. As soon as they had been diagnosed with HPV, some of the patients were haunted by the suspicion that their spouses were cheating on them.

Well, they’re usually suspicious of their spouses and the love that they felt for each other before is gone. I mean they keep wondering how their spouses had gotten the infection. (Participant 8, doctor).

On the other hand, the person who transfers the infection has a guilty conscience and constantly blames him/herself for having engaged in high-risk behaviors. Some individuals become obsessive and suffer from hypochondria or fear of transferring their disease or spread of the disease to other parts of their body.

I couldn’t help worrying. I thought if I drank water from a glass and then my brother drank from it, he would get sick too. Or when I touched the water taps, I was worried they would touch them too and get infected. (Participant 15, patient).

##### Struggling with mental pressure

All of the patients had experienced mental pressure and most of them had cried after being diagnosed with the infection. Some had contemplated taking revenge on their spouses or the society or committing suicide—some had actually attempted suicide. Recurrent nightmares, insomnia, turning to superstitions, and threatening their spouses with cheating were among the issues which the participants mentioned.

I wanted to kill myself, to put a string around my neck and suffocate myself. I was fed-up with my life. (Participant 16, patient).

The patients’ reactions to the news that they were infected included indifference to satisfaction of their basic needs, lack of motivation, depression, emptiness, and meaninglessness of life and its interests.

The whole world has turned black for me. I have no hope for my life. I just want to die. (Participant 1, patient).

Some of the patients were suffering from appetite loss, lethargy, failure at school or work, less interest in life, and indifference to their appearances. The findings of the study showed that all the patients were displaying obsessive behaviors, including obsession about hygiene, repeated examination of their bodies, repeated visits to their therapists, and asking repeated questions.

One of the patients said, ‘I wash my hands ten times a day and have separated my dishes from the rest of the family. (Participant 1, counselor).

Many of the patients mentioned that they were obsessive about examining their bodies because they were worried about recurrence of their genital warts, increase in their number, or spread of the infection to other parts of their bodies, including the throat. One of them said, “*I’m always holding a mirror, checking my genitalia*” (Participant 6, patient).

Following their diagnosis with HPV, many of the patients limited their social interactions, became reserved, or decided to stay single. Some patients explained that they opted to stay single because they did not want to be despised and reprimanded or enter a marriage with an uncertain future because of their sexually transmitted disease. Some of the other patients had quit their jobs, decreased or ended their contact with their friends and family, or reduced their social interactions. Some of the infected had stopped having sex temporarily following their diagnosis and some of them had abandoned sexual relationships even though a few years had passed since their infection.

My sexual relationships were down to zero. I could not be with anyone for the first three months. (Participant 2, patient).

To avoid telling the truth about how they had been infected, the patients named swimming pools, sauna, or their friends’ clothes as the sources of their infection.

#### Trying to maintain resilience

Trying to maintain resilience consisted of the concepts of “seeking help” and “making the best of a bad situation.”

##### Seeking help

All the participants referred to the need of the infected for support from friends and family, psychologists, psychiatrists, and healthcare providers.

At some point, you would give anything just to be able to talk to someone. (Participant 2, patient).

To satisfy their need for emotional support, the infected seek support from the people around them or healthcare providers. *I was so scared and crying. It was 10 at night. I called my sister and begged her to come and help me. I was just crying.* (Participant 6, patient).

The participants also mentioned that it is very important that healthcare providers consider the psychological needs of the infected in their interactions with them. Also, all of the patients searched for information on the Internet to increase their knowledge of the disease and learn about other people’s experiences of their infection. In addition to the Internet, the patients tried to get information from their friends, other patients, and healthcare providers.

##### Making the best of a bad situation

One of the coping strategies of the patients was increasing their resilience. This strategy included such behaviors as reassuring their spouse/sexual partner, ignoring their family’s critical attitude, turning to religion, keeping their infection secret, hoping that things get better with time, accepting the situation, and keeping themselves busy with other things. One of the participants said, *My mom and dad constantly scolded me. So I just decided to ignore them. I didn't care anymore?* (Participant 9, patient).

Some of the patients stated that after their diagnosis with HPV, they tried to reduce their stress by turning to God and other sources of spirituality. Secrecy was a strategy employed by all the patients to avoid stigma, implications about how they had been infected, and the negative reactions of the people around them.

Even now, nobody knows I’m here [at the clinic]. I just told them I was going out. I don’t want anyone to know. (Participant 5, patient).

Sometimes, the patients conclude that their concerns, which are the source of their stress in life, are endless and since they cannot eliminate them, they should wait for time to improve the situation. Some of the patients said that their strategy was to accept the situation or just take it easy. Some of the other patients kept themselves busy with other things, e.g. housework and playing music, to keep their minds off their infection.

I try to be in my workplace so I can keep busy and not think about it. (Participant 8, patient).

Changing lifestyle was another strategy used by the patients. They used traditional medicine, improved their nutrition and sleeping habits and exercised to strengthen their immune system in the hope of eliminating the virus from their body.

I’ve decided not to eat any more fast food or processed foods. (Participant 10, patient).

Some had quit high-risk sexual behaviors and some others had taken the vaccine and underwent screening to avoid contracting other viruses and prevent their infection from developing into cancer.

### Consequence: oscillation between tension and tranquility

The findings of the study showed that the consequence of the patients’ action-interaction in relation to stress in life—their main concern—was “oscillation between tension and tranquility.” This category consists of the concepts of “mental exhaustion” and “achieving relative tranquility.”

#### Mental exhaustion

As mentioned above, because of their fear of being stigmatized, some of the interviewed patients did not seek help from others and their isolation in the family made their situation more complicated. Moreover, the inefficacy of some of their coping strategies made them feel incompetent, inefficient and self-alienated and undermined their self-confidence and self-esteem.

What good can two sick people [referring to himself and his wife] have for the society? Why should we live? (Participant 16, patient).

Stress in life, lack of awareness, inconsistency in the content available on the Internet, and contradictory information given by healthcare providers confuse the patients. Some of the patients were afraid to have sexual relationships again and the fear lasted in some cases.

I’ve started to hate having sex. I don’t even want to think about it. When my husband approaches me, I feel disgusted, I’m scared [she is crying]. (Participant 6, patient).

Unsupportive behaviors on the part of the patients’ friends, family and care providers, including putting them under pressure, rejecting them, and scolding them, were disturbing to the patients. Having genital warts, being treated by others with scorn, and being criticized by their families created feelings of frustration and shame in the patients. The participants stated that, despite the coping strategies which they applied, some continuing concerns filled their lives with apprehension. Some of the participants used the phrase “like hell” to describe their lives.

I’m really nervous. We haven’t had any sexual relationships with each other for many years now. This is not living. This is being in hell. When you’re always stressed about if you’ll get cancer or not, your life will be like hell. (Participant 1, patient).

Tension in marital relationships led to many issues, ranging from frequent quarrels to sexual dissatisfaction.

#### Achieving relative tranquility

Certain coping strategies, including adopting a spiritual lifestyle, seeing a psychologist, and taking tranquilizers, were found to have created relative tranquility in the patients.

I wanted to go to a place where I could have peace. Visiting Shahcheragh [a holy shrine] made me feel so calm. (Participant 17, patient).

A few of the participants mentioned that when some of their concerns, including the risk of their transferring the infection to their families, safe methods of having sex and their chances of having cancer, were eliminated, they felt less stressed. They stated that they could laugh again like before, resumed their professional activities, attended to their household affairs, and took up their social activities again.

Before that, I didn’t feel like taking my daughter to her ballet class and this had a terrible effect on her spirits. I hit my son a lot. I didn’t cook. But now I talk to my kids; we laugh; I take them to their classes; I cook. (Participant 5, patient).

Some of the patients mentioned that they felt less stressed after they obtained the right information about HPV, including that facts that only the high-risk types of the virus can cause cancer in the long run which can be detected in the early stages of development through screening, the virus can be eliminated by the immune system, genital warts are treatable, infections caused by the low-risk types of the virus are benign, and HPV had a high prevalence rate.

When I was told that I won’t transfer it my family, I felt so calm; it was such a relief. (Participant 7, patient).

The above-mentioned strategies helped the patients recover some of their tranquility. However, the persistence of some of their concerns kept the patients oscillating between tension and tranquility. The domain of consequences is a dynamic spectrum affected by contextual and intervening conditions and each patient can be placed at a certain point on the spectrum.

I tried … I mean I have to accept it and stop thinking about it. Even though it’s been more than a year now, I still can’t help thinking about it; it’s always on my mind … I keep saying, ‘well, what can I do?’ … Then I tell myself to forget it and this cycle just goes on. (Participant 18, patient).

## Discussion

“Trying to maintain resilience in the absence of psychological security” was the theory which emerged from the analysis of data in the present study. Management of HPV is adversely affected by such factors as false beliefs about the infection [24], concern about controlling the symptoms, the risk of developing cervical cancer, fear of transferring the infection to one’s embryo or sexual partner, recurrence, mortality and incurability of the disease [14, 15, 19, 25], all of which are confirmed by the findings of the present study. According to a study, in addition to women infected with the high-risk types of the virus, women with the low-risk types also worry about developing cervical cancer. They attribute their concern to the possibility of contracting the high-risk types of the virus [17]. On a similar note, the results of the present study show that the wrong information provided by some healthcare providers and the social media about the likelihood of a low-risk HPV changing to a high-risk HPV, the patients infected with the low-risk types of the virus are worried about developing cancer too.

Another study reports that women with abnormal cervical cytology are worried about the impact of the disease on their fertility [26]. However, in the present study, the patients (both man and woman) were concerned about the possibility of the infection being transferred to their embryos or babies during pregnancy or at birth. The discrepancy between the research results may be due to the differences between the participants of the two studies. In the former study, the participants consisted of women with abnormal cytology, while the participants in the present study did not have any signs of pathologic cellular changes.

The results of the present study also show that the concerns of the infected include disruption to family relationships and tension in marital relationships after testing positive for HPV, which agrees with the findings of other studies [15, 27].The participants attributed the disruption to family relationships to taboos about unlawful relationships, suspecting one’s spouse of having affairs, and the contagious nature of HPV.

In the present study, many of the participants had experienced being judged and disrespected by their families, the healthcare system, and the whole society, which added to their tension. Other studies have reported similar results [15, 28]. The number of studies conducted on men infected with HPV is quite small. One study reports that women are more likely to experience the stigma associated with HPV than men are [28]. The participants in the present study affirmed that, as a result of sociocultural beliefs, the issue of stigma is more serious for single women than single men; however, with regard to married men and women, the difference was not significant. The discrepancy between the results of the two studies can be attributed to differences between their methodologies: the former study is a quantitative work, while the present study has used a qualitative approach and includes an in-depth analysis of the patients’ experiences. Also, in the present study, the patients stated that the emotional and informational support of their friends, families, and healthcare providers raised their spirits. Likewise, other studies report that emotional/informational support and positive social interactions improve the psychological well-being and treatment results of patients with HIV [29] and reduce their levels of depression [30].

The greatest sociocultural barrier to sex education in Iran is the taboos about sexual relationships and discussing matters related to sex. In addition, ignorance in most families about sexual matters results in teenagers’ experiencing their first sexual relationships with little awareness of sexual health [31]. In the Iranian-Islamic culture, families and the society are reticent about sexual matters, including sex education [32], as confirmed by the views of the participants in the present study. These obstacles can be overcome with the support of organizations in the public and private sectors and policy-makers toward empowering families and teachers to raise teenagers’ sexual health awareness [31].

Patients use the social media to increase their knowledge about their infection and to find doctors. The media also enable patients to interact with other patients as well as doctors. However, it is hard to evaluate the trustworthiness of these sources of information and misleading information can be harmful to patients [33]. Studies confirm the significant role of media in alleviating the adverse psychosocial effects of HIV [34]. The findings of the present study show the same outcome with patients infected with HPV. However, the manner of transferring information and the quality of the information provided are very important: only high-quality content on media can make an impression on people’s minds; information of low quality will repel its audience and aggravate the current discriminatory behaviors in the society [34]. The participants in the present study also referred to the role of media, but much of the content obtained by the patients came from weblogs and personal accounts on Instagram as the national media in Iran do not usually address HPV.

The results of a study show that the process of organizing data on women infected with HPV is complex, non-linear, and irregular. Some women make immediate responses to positive diagnosis results, while others’ responses appear over time. Immediate responses include shock and confusion due to lack of knowledge. The infected start to search for information on the Internet and from healthcare providers. Learning about the consequences of HPV—genital warts and cancer—changes their shock to fear [17]. Similarly, in addition to the above, the results of the present study show that the patients’ repeated visits to healthcare providers and receiving contradictory information from them and the social media add to their fear and confusion. In addition, following the shock of their realization that they were infected, the patients in the present study wanted to distort the truth about their infection, especially about how they had been infected, which is the result of the dominant cultural-religious beliefs in Iran.

Patients diagnosed with HPV also experience shame, stigma, regret, self-reproach [19], disruption to their relationships, negative impact on their social relationships, inclination to hide their test results, obsession with cleanliness, preference for distant clinical interactions e.g. using e-mail to receive their test results, fewer social interactions, and disruption to their sexual performance [13, 14, 27]. Women patients’ negative responses at the time of their diagnosis and thereafter adversely affect their sexual behaviors and marital adjustment. Sexual dissatisfaction and unsafe attachment undermine couples’ marital adjustment [35], which is consistent with the results of the present study. According to a study, after being diagnosed with HPV, women display negative feelings, including anger, confusion, depression and shock, while men rarely respond in this manner [28]. In the present study, however, the man patients had experienced negative feelings too. The discrepancy can be attributed to the differences between the methodology of the former study (quantitative) and that of the present study (qualitative).

Fear of stigma causes the infected to adopt the strategy of hiding the truth [14]. In the present study, many of the patients had hidden their infection from the people around them (except for their spouses and, in some cases, first-degree relatives) for fear of being labeled. The results of a study in Iran show that personal responses to testing positive for HPV include marital conflicts, inclination to divorce, concealing one’s infection from one’s sexual partner and not taking one’s infection seriously [15]. On the contrary, in the present study, most of the patients, both man and woman, had informed their spouses or sexual partners of their infection, but, to avoid stigma, had kept it hidden from their other family members and friends. Many had informed their spouses to prevent infecting them. Also, none of the patients in the present study showed signs of indifference to their infection after having been diagnosed with HPV. One of the concerns of the patients in the present study was having a guilty conscience about transferring their infection to their sexual partners, a finding not reported by other studies. This difference can be attributed to the fact that, unlike other similar studies, the participants in the present study consisted of both genders. In the present study, the man patients felt guiltier about infecting their sexual partners, which can be because men are more likely to be engaged in unlawful relationships [36, 37] and most of the married women in the present study had been infected by their husbands.

In a study in the U.S., the woman participants were found to have accepted their infection with HPV as a chronic disease due to its uncontrollable nature and suppressed their thoughts in an attempt to adapt to the uncertain conditions of their disease. However, when they were accidentally exposed to information, their attempts at thought suppression were futile [17]. This finding agrees with the results of the present study where the participants used such strategies as seeking help, increasing resilience and changing lifestyles, besides thought repression, to adapt to their situation. The results of a quantitative study show that the most common mechanisms employed by individuals with clinical symptoms of HPV are planning, active adaptation, self-reproach, seeking emotional support, positive reframing, and acceptance; the least frequently applied mechanisms are turning to religion and drug abuse [38]. In the present study, however, turning to religion as a coping strategy was one of the most common adaptation mechanisms used by the patients. The reason for this discrepancy can be the cultural differences between the two countries and the prominence of religion in Iranian societies [39]. The results of studies conducted in Iran indicate that religious and spiritual practices improve the psychological well-being and quality of life of patients with chronic diseases, e.g. multiple sclerosis [40], reduce mental tension in patients who receive dialysis [41], and facilitate adaptation [42] and self-care [43] in patients with cancer.

As literature reviews show, so far few studies have used a quantitative approach to address the adaptation of woman patients with HPV over time [20, 44]. According to one quantitative study, 12 months after their diagnosis, patients with HPV still reported certain levels of psychosexual anxiety which, as the authors explain, may have been the result of their responding to the questionnaire—response bias [44]. In the above-mentioned study, women with a history of HPV who tested negative at the time of the study still had psychosexual concerns due to their fear of recurrence of their infection in the future. The authors conclude that the subjects may have had reasons for their psychosexual concerns 12 months after diagnosis, but the quantitative design of the study does not allow for examining those reasons, thus they suggest qualitative research for investigating why the concerns of the infected persist [44]. Studies report that the greatest long-term concern of women infected with HPV is that their disease is infectious [13, 44]. In the present study, also, the participants mentioned that their concerns continued over time. According to a quantitative study, the psychosocial adaptation of women diagnosed with HPV is dynamic in nature [20]. Likewise, the coping strategies of the patients in the present study changed according to their conditions and the patients were in a state of oscillation between tension and tranquility. The authors of the above-mentioned study state that the infected can achieve sexual and psychological adaptation after 6 months, but in terms of health care orientation, more time is need. One of the limitations of this study is that it does not address the coping strategies and conditions of the patients; also, due to its quantitative methodology, it lacks an in-depth analysis of the patients’ experiences [20].

Studies show that treatment of genital warts and initiation of sexual relationships can reduce anxiety in patients with HPV and facilitate their psychosocial adaptation [20, 45]. In the present study, the anxiety levels of the patients decreased over time and many of them reinitiated their sexual relationships. But, as the patients stated, due to their various concerns and the impact of their conditions, the quality of their sexual relationships was not the same as it was before their infection. In the present study, the single men had reinitiated their sexual relationships while practicing safe sexual behaviors, but the married men who were rejected by their wives had not been able to establish their former sexual relationships.

In summary, the theory which emerged from the findings showed that the patients’ main concern was life stress. The stigma of having STDs and the society’s poor awareness about HPV affect the patients’ perception of their disease and how they and their families react to their testing positive for HPV. The strategies used by the patients to cope with their stress were emotional confrontation and maintaining resilience. The outcome of these interactions and strategies was oscillation between tension and tranquility. Identification of the patients’ concerns and the conditions which affect their adaptation can help healthcare policy-makers and providers develop and implement supportive programs to improve the quality of life of the infected.

One of the strengths of the present study is its use of the grounded theory approach which allowed for developing a theory based on the cultural and social context of patients infected with HPV. In addition, the focus of most previous studies in this field has been women, while the participants in the present study consisted of both man and woman patients with HPV, healthcare providers, and spouses of patients. The study limitations also comprised failure to find homosexuals of either gender despite making great efforts to observe maximum variation in selecting the key participants and failure to interview with only bisexual participants. Another limitation of the study might be the gender difference between the woman interviewer and man participants. This gender difference might affect the men’s responses to some questions.

## Conclusion

In the present study, the researchers used the grounded theory approach to describe the adaptation process of patients with HPV. The results showed that adaptation to being diagnosed with HPV is a dynamic and context-dependent process. In this process, patients tried to maintain resilience in the absence of psychological security. Among the influential factors in the dynamics of this process and oscillation between tension and tranquility were a context of stigma and ignorance and paradox in support. The findings of the present study stress the need for the establishment of a standard approach to treatment of genital warts in clinical centers, especially in the private sector, allocation of capable counselors to clinics for patients with HPV, and eliminating the stigma associated with the infection in the healthcare system and the society.

In addition, an understanding of the patients’ perception of their disease is essential to development of effective educational interventions whose aim is change the patients’ perspective on their situation and improve their recovery. Also, in view of the low level of public awareness about HPV and sexual health and provision of wrong information to the infected, it is recommended that educational interventions focus on the patients’ concerns. Timely interventions, including counseling services to alleviate the psychosexual tension of testing positive for HPV, can improve the patients’ adaptation and protect them from the potential harms of the infection. Accordingly, it is suggested that future studies be centered around developing consultative-educational programs based on the needs of patients with HPV and evaluate the effectiveness of those programs on the quality of life of the patients.

## Data Availability

All data generated or analyzed during this study are included in this manuscript. The MAXQDA file is not publicly available due to participant information confidentiality but is available from the corresponding author on reasonable request.

## Abbreviations

HPV: Human papillomavirus
HIV: Human immunodeficiency viruses
STDs: Sexually transmitted diseases
