# Correction to: Adaptation of patients diagnosed with human papillomavirus: a grounded theory study

**DOI:** 10.1186/s12978-021-01288-4

**Published:** 2021-11-26

**Authors:** Narjes Nick, Camellia Torabizadeh, Mehdi Ghahartars, Roksana Janghorban

**Affiliations:** 1 Department of Nursing, School of Nursing and Midwifery, Community Based Psychiatric Care Research Center, Shiraz University of Medical Sciences, Shiraz, Iran; 2 Molecular Dermatology Research Center, Shiraz University of Medical Sciences, Shiraz, Iran; 3 Department of Midwifery, School of Nursing and Midwifery, Community Based Psychiatric Care Research Center, Shiraz University of Medical Sciences, Shiraz, Iran

## Correction to: Reprod Health (2021) 18:213 10.1186/s12978-021-01264-y

After publication of this article [1], the authors reported that in the pdf-version of the article the statement in the Funding-section was incorrect. It should have read “Shiraz University of Medical Sciences provided funding for the research. The funding body had no role in the design of the study, collection, analysis, or interpretation of data. The funding body also had no role in the writing of the manuscript.”

The original article [1] has been updated.
